# Mind the gap: an attempt to bridge computational and neuroscientific approaches to study creativity

**DOI:** 10.3389/fnhum.2014.00540

**Published:** 2014-07-24

**Authors:** Geraint A. Wiggins, Joydeep Bhattacharya

**Affiliations:** ^1^Computational Creativity Laboratory, School of Electronic Engineering and Computer Science, Queen Mary, University of LondonLondon, UK; ^2^Department of Psychology, Goldsmiths, University of LondonLondon, UK

**Keywords:** creativity, neuroscience, psychology, computational modeling, methodology

## Abstract

Creativity is the hallmark of human cognition and is behind every innovation, scientific discovery, piece of music, artwork, and idea that have shaped our lives, from ancient times till today. Yet scientific understanding of creative processes is quite limited, mostly due to the traditional belief that considers creativity as a mysterious puzzle, a paradox, defying empirical enquiry. Recently, there has been an increasing interest in revealing the neural correlates of human creativity. Though many of these studies, pioneering in nature, help demystification of creativity, but the field is still dominated by popular beliefs in associating creativity with “right brain thinking”, “divergent thinking”, “altered states” and so on (Dietrich and Kanso, 2010). In this article, we discuss a computational framework for creativity based on Baars’ Global Workspace Theory (GWT; Baars, 1988) enhanced with mechanisms based on information theory. Next we propose a neurocognitive architecture of creativity with a strong focus on various facets (i.e., unconscious thought theory, mind wandering, spontaneous brain states) of un/pre-conscious brain responses. Our principal argument is that pre-conscious creativity happens prior to conscious creativity and the proposed computational model may provide a mechanism by which this transition is managed. This integrative approach, albeit unconventional, will hopefully stimulate future neuroscientific studies of the inscrutable phenomenon of creativity.

## Introduction

In recent years, the scientific study of creativity has burgeoned (Vartanian et al., 2013). However, the problem has proven to be inscrutable, and the various different approaches have not made as much progress as initially expected. Part of the reason for this is without doubt the as-yet-unclear specification of what the key questions are; part is without doubt the sheer complexity of the problem. But part, we claim, is also down to the lack of a suitable integrated framework within which the various approaches (neuroscientific, psychological, computational, philosophical, etc.) may collaborate. The aim of this paper is to provide a hypothetical framework for research, built around a key idea, which itself is a combination of ideas from cognitive psychology and communications engineering. These ideas in their own right are far from novel; their combination, however, affords a mechanism for creativity which yields testable predictions of creative and other behavior.

The argument is structured as follows: we begin by deconstructing the social notion of creativity, to the point at which it may be possible to study it in a reductionist context, and give a review of earlier attempts to do so, with an emphasis on the neuroscientific. Next, we describe a proposal for a cognitive architecture that supports creative production, based on extant models of efficient memory and information processing. We then describe some potential neuroscientific approaches to testing the model. Ultimately, we propose that our tripartite combination of neuroscientific, behavioral and computational methods is an example of the kind of relatively sophisticated methodology required to address what is, after all, one of the fundamental questions of humanity.

## Creativity—what is it?

Creativity is notoriously difficult to study. Even the meaning of the word itself is confounded by subjective value judgments, and the problem of evaluating created artifacts rigorously is difficult enough to deserve its own section. Therefore, it is appropriate to begin with a deconstruction of this troublesome concept, with a view to specifying what is and what is not amenable to scientific enquiry. A key part of this endeavor is demystification: our claim is that the perception of creativity in an action is relative to creator, observer, and their social context. That relativity has, in the past, led to a significant Romanticization of creativity in some societies, which, we believe, can obscure its true nature.

We begin from the premise that creativity is fundamentally a property of a process (as in “a creative act”). The exhibition of that property may then, in common parlance, be transferred to the organism or machine that is executing the process (as in “a creative person”) or its product (as in “a creative novel”). However, we eschew these less precise usages here.

We are not alone in focusing on process. Cognitive and behavioral psychologists have been investigating the creative process for almost a century. Wallas (1926) suggested that the mental process of creativity with four observably distinct stages: preparation, incubation, illumination and verification. Guildford’s (1967) proposed the creative process is often characterized by divergent thinking, a break from the previous, most obvious type of thinking, followed by convergent thinking, a narrowing process of choosing the “best” idea among the available options. Getzels and Csikszentmihalyi (1976) described mental states during occurrence of the process, the best known aspect of which is the state of “flow” experienced by a creator during a process of creativity. Koestler (1964) proposed a vague but convincing process of “bisociation” of cognitive structures, which has recently formed the basis of a successful computational research project (Dubitzky et al., 2012). Boden (2003) gave a general cognitivist theory of creative conceptualization and process, which has been formalized as a mathematical “Creative Systems Framework” by Wiggins (2006a,b), with a view to computational study. Finally, a significant series of work from the past decade and a half, which remains somewhat unfamiliar to psychologists and neuroscientists working on creativity, is that of Jürgen Schmidhuber (2010), whose theory relates a notion of intrinsic reward, derived from a rate of learning, to the discovery of new patterns that are compressible. This theory relies on ideas from Information Theory, to which we return in the later section.

One advantage of taking the process view is that it begins to demystify creativity itself, simply because value is normally attributed to a product or artifact only after its achievement (Boden, 1998); thus, by studying the creativity before the artifact is judged, we can blow away some of the Romantic fog. Further, the (perceived) value of a product is never constant, but is dependent on society, places, groups, time (e.g., El Greco (1561–1614) was ridiculed for his style of painting during his life-time, but now is considered to be one of the foremost Renaissance painters). So the process of valuation is very subjective, and constrained by socio-cultural factors, therefore defying empirical objectivity (Schaffer, 1994). Thus, success and the associated social pedestals on which “great creators” are placed, become quite irrelevant: it is the attempt not the product that counts in the first instance. Thus we defuse, to some extent, the debate between what researchers call “big-C creativity” and its lower case counterpart (Kaufman and Sternberg, 2006). Having disposed of these highly subjective and emotive judgments, we can better identify creativity in a wide range of activities, and therefore be in a better position to understand it as a whole.

Having settled on the process view of creativity, we must consider what is executing the process. Since there is evidence that some animals (e.g., corvids, pigeons) can exhibit complex cognition including creative tool construction and insight problem solving (Epstein et al., 1984; Emery and Clayton, 2004; van Horik et al., 2012; Jelbert et al., 2014), it would be inappropriate to restrict study to humans (Kaufman and Kaufman, 2004). The new research field of Computational Creativity has explicitly drawn creativity into the purview of Artificial Intelligence (Colton and Wiggins, 2012), and therefore it seems appropriate to consider the possibility of non-biological organisms exhibiting creativity also. There is, of course, a debate about whether computers can be properly called creative (Cohen, 1999); however, to rule them out *a priori* would be to prejudge its outcome. The benefit of this inclusive approach is that it admits study by simulation, in the same way that computational linguistics has done for the study of linguistic cognition. Above all, the computational approach forces us to formulate our theories to an extreme level of detail, and allows us to test them to destruction in ways that would be ethically questionable if applied to biological organisms, yielding a very powerful method. Ultimately, it does not matter if one believes that the computer is “really” creative, or if it merely appears to be so (Turing, 1950): the methodological value remains.

The next distinction to make is between what we term, on one hand, spontaneous creativity and, on the other, creative reasoning. The former corresponds with the moment of illumination in Wallas’ theory (Wallas, 1926), the “Aha!” moment. It is often preceded by Guildford’s (1967) two searching phases. It is the event that causes awareness of an unanticipated outcome in a creator, where “outcome” is interpreted broadly: what enters awareness need not be a solution to a problem, for example, but might instead be the method by which to find one. This is best understood in contrast with creative reasoning, which is the deliberate, conscious application of reasoned construction steps in producing an artifact that will ultimately be deemed creative. An example of the latter is the jobbing song-writer, who has been commissioned to write the theme tune for a radio program, by a certain date. The song-writer cannot simply wait for spontaneous creativity to appear (or she will be soon in breach of contract); rather, she must apply expert knowledge of her domain to come up with something novel. As one learns in undergraduate music courses, it is possible to do this by means of considered application of musical rules, though merely generating musical structures in this way does not guarantee a satisfactory musical experience at the end; skill is required. Creative reasoning, then, is the deliberate application of construction steps in the creative process.

Having made this distinction, we note that many creative processes will consist of a combination of both kinds of creativity. Wolfgang Amadeus Mozart described his own creative process in this way, as a cycle of spontaneous creativity and then creative reasoning (in this case, selection):

“When I am, as it were, completely myself, entirely alone, and of good cheer—say traveling in a carriage, or walking after a good meal, or during the night when I cannot sleep; it is on such occasions that my ideas flow best and most abundantly. Whence and how they come, I know not; nor can I force them. Those ideas that please me I retain in memory, and am accustomed, as I have been told, to hum them to myself.

“All this fires my soul, and provided I am not disturbed, my subject enlarges itself, becomes methodized and defined, and the whole, though it be long, stands almost completed and finished in my mind, so that I can survey it, like a fine picture or a beautiful statue, at a glance. Nor do I hear in my imagination the parts successively, but I hear them, as it were, all at once. What a delight this is I cannot tell! All this inventing, this producing takes place in a pleasing lively dream. Still the actual hearing of the toutensemble is after all the best. What has been thus produced I do not easily forget, and this is perhaps the best gift I have my Divine Maker to thank for” (Holmes, 2009, pp. 317–318).

Wiggins (2012a) expounds this argument in more detail. For the current purpose, the distinction serves to highlight a difference between two modes of creativity, of which most humans have experience, and which may or may not demand different mechanisms and/or neural loci, thus entailing an interesting set of research questions.

## Creativity—why is it there?

An important question in the scientific study of creativity is, “why and how should such a faculty evolve?” This is not a question that is open to empirical science, for obvious reasons. However, to proceed without considering the evolution of such an important cognitive faculty, and without proposing at least one pathway by which mechanisms predicted by one’s theory might evolve, is to admit the possibility of fiction. To answer this question requires careful teasing apart of the various meanings of the C-word, and of the value judgments associated with them, and also of the different ways in which evolution can take place. First, let us briefly distinguish between biological and social evolution.

In the biological case, one might argue for creativity as an outcome of evolutionary processes themselves: the very existence of humankind, for example, might be deemed an act of creativity. However, it seems to us that attributing the property of creativity to a process which has no definable agent^1^ is less than meaningful. Therefore, we draw a line between the process of evolution (which is, perhaps, more serendipitous than creative, in the final analysis), and the processes applied, one way or another, by agents or groups of agents in the world. Thus, neither the scarab beetle “creating” its ball of dung, nor the evolutionary chance sequence that gave rise to it, falls within our definition of creativity.

In the social case, on the other hand, it is hard to deny the existence of creative evolution. Indeed, it is impossible to achieve a situation in which an individual can create a concept that persists through arbitrary time, but is also unknown to all parts of society. Given this basic truth, and that transmission of information between biological organisms is imperfect, and that no two biological individuals have exactly the same background knowledge, we are left with a situation somewhat analogous to evolution, which is the analogy amplified in the study of so-called “memetics” (Dawkins, 1989; Blackmore, 2000). It is not necessary to go to such extremes to see a process analogous to evolution working in society: the development of Western music since the 13th century is a paradigmatic example.

The key question, though, is, “what evolutionary pressure would favor the capacity for individual creativity?” In a later section, after Wiggins (2012b), we propose that creativity results from a general cognitive mechanism of adaptive prediction, which allows organisms to manage the world more effectively by predicting the immediate future, based on experience, rather than merely reacting to events in the world. Other evidence for the importance of anticipation in humans is given by Huron (2006). Thus, as above, we are drawn to the suggestion that creativity is not a special faculty in its own right, but a combination of properties which contribute to evolutionary fitness. These properties may contribute to fitness for creative or non-creative reasons in the individual, or to sexual (or other social) selection, in the group, or both.

## Creativity—how can we measure it?

If creativity is hard to define, it is even harder to measure. There is no single measure or method that can adequately capture the multifaceted nature of creativity; in fact, well over a hundred measures have been developed and applied to this purpose (Plucker and Makel, 2010). Here we briefly mention some of the most commonly used ones.

The current empirical research on creativity was spearheaded by Joy Paul Guildford who in his Presidential address in 1950 suggested to the American Psychological Association that creativity, though an elusive construct, could be psychometrically studied. Over the following decade, Guilford and his team developed the Divergent Thinking Test (DTT): for any problem, there exist many possible solutions which may qualitatively differ from each other. For example, in the “unusual uses” test, an example of a DTT, participant is asked to list many different uses for a familiar object (e.g., brick, paper-clip), and the responses are coded by independent raters to judge: (i) fluency (the number of responses: the more ideas a creative person can come up with, the greater the chance that some of it will be useful); (ii) originality (responses that are less-frequently reported by others: a creative person produces ideas that are unique); (iii) flexibility (number of responses falling into distinct categories: a creative person is flexible, i.e., can break away from habitual mode of thinking); and (iv) elaboration (the detailed nature of responses: a creative person has a detailed plan). The concept of divergent thinking subsequently led to the Torrance tests of creative thinking (Torrance, 1974), the most widely used paper-and-pencil test of creativity.

Another influential test of creativity, the remote associate test (RAT), is based on the concept of associations and convergence: creativity involves combinations of two remotely associated concepts in a novel and convergent way (Mednick, 1962). Here, a participant is given a list of word triplets (e.g., paper, stone, fire), and asked to produce a target word (wall) that makes three valid compound words (wallpaper, stonewall, firewall). As each triplet is likely to have a fixed solution, this test, unlike the DTT, does not require any subjective judgment.

Finally, the Consensual Assessment Technique (CAT) was developed specifically to assess creativity perceived in finished artifacts (Amabile, 1996). A group of experts in the domain of the artifacts are invited to make judgments about the creativity of the artifact in question, including discursive remarks explaining their judgment in detail. Statistical analysis is used to ensure that only the judgments where the experts are unanimous are used, thus reducing subjectivity. The experts’ remarks can be unified to develop an account of their reasons for their judgments.

Further, creativity is often associated with cognitive insight, the sudden understanding of the solution to a problem without any conscious forewarning (the “Aha!” experience; Wallas, 1926). Researchers either use classical insight problems to characterize creativity (Metcalfe and Wiebe, 1987) or distinguish non-creative performances from creative ones based on subjective experience of insight (Jung-Beeman et al., 2004).

Though these kinds of test batteries are very useful to study little-c creative processes in a laboratory setting, the possible relationships between the scores on these tests and big-C creativity are not conclusive (Sawyer, 2012). Yet it is increasingly accepted that creative thinking, even at the highest level, is not mysterious, but rather composed of standard cognitive processes, including problem solving (Weisberg, 2006). Our approach, therefore, is to consider mechanisms and evaluation methods that can account for both big-C and little-c creative behavior, and anything in between.

Computational cognitive modeling gives us a substantial advantage in the context of evaluation, because theories that are computational are no longer static objects that serve only as thought experiments, but active ones capable of action on information, or even, given a robot, in the physical world. That is to say, a computational theory can be implemented, as a program, and run, and its behavior studied, in comparison with human behavior, or as a separate creative “organism” in its own right. This approach is the keystone of the hierarchical modeling strategy described by Wiggins (2011), which we propose to use here. First, we offer a brief overview of the neuroscientific studies of creativity and some possible limitations.

## Creativity—where in the brain?

### Neuroscientific findings on creativity

Over the last decade, a substantial body of research has been published on neuroscience of creativity. The literature can broadly be divided into three groups: (i) studies using divergence (DTT) or convergence (RAT) tests; (ii) studies on “Aha!” insight; and (iii) studies using professionally creative individuals like musicians or artists. A diverse range of neuroimaging techniques, from correlational methods like EEG, MEG and f-MRI to causal stimulation methods like transcranial magnetic stimulation (TMS), transcranial direct current stimulation (tDCS) have been used. In addition to these functional neuronal correlates, researchers have also been interested in establishing link between creativity and specific brain structure(s) by studying patients with brain injury (lesion studies) and by adopting latest structural neuroimaging measures including diffusion tensor imaging (DTI) and variants of magnetic resonance spectroscopy (MRS). It is beyond the scope of this paper to provide a comprehensive review of neuroscientific findings, and we refer the readers to some excellent recent review articles (Arden et al., 2010; Dietrich and Kanso, 2010; Jung et al., 2013). Nevertheless, we briefly mention here some of the key findings.

For example, f-MRI studies of DTT suggest widespread activations over prefrontal cortex but without any clear hemispheric lateralization (Howard-Jones et al., 2005; Fink et al., 2009a). EEG studies do not indicate any consistent hemispheric lateralization nor any consistent changes in terms of alpha power (8–12 Hz) (Dietrich and Kanso, 2010); however, a recent review points towards a systematic effect of alpha increase for the creative generation of ideas (Fink and Benedek, 2012). Neuroimaging studies of insight problem solving suggest that, during sudden comprehension of the problem, multiple brain regions activate, including the anterior cingulate, hippocampus, anterior superior temporal gyrus, right prefrontal cortex (Dietrich and Kanso, 2010). EEG studies provide temporal dynamics of the neuronal activations underlying cognitive insight during convergent thinking: enhanced gamma band power (*>*30 Hz) in the right frontal cortex is observed 300 ms before the subjective “Aha!” moment while solving remote associate problems (Jung-Beeman et al., 2004; Sandkuhler and Bhattacharya, 2008), and this is interpreted as the sudden conscious availability of the target solution words. Remote EEG brain waves up to 8 s before the insightful solution are also reported in participants while solving insight problems (Sheth et al., 2009). Further, EEG alpha (8–12 Hz) power is found to be associated with mental states prepared for insight (Kounios et al., 2006). Brain stimulation studies suggest that performance on RATs could be enhanced by applying electrical stimulation to various brain regions, i.e., the dorsolateral pre-frontal cortex (dLPFC; Cerruti and Schlaug, 2009), right anterior temporal lobe (Chi and Snyder, 2011). Altogether the neuroscientific studies have adopted the task paradigms (i–ii), of divergent and convergent types, which are grouped together under little-c creativity.

The neural correlates of creative processes associated with big-C creativity could be investigated by studying brains of creative professionals in action; so far only a few studies have investigated creativity-related (like improvising music, composing an artwork) brain activity in samples of performing artists like visual artists (Bhattacharya and Petsche, 2005), musicians (Limb and Braun, 2008), dancers (Fink et al., 2009b). During mental composition of drawings, professional artists showed greater long-distance synchronization between multiple brain regions in low frequency neuronal oscillations (<4 Hz), suggesting a more emphasized top-down processing (Bhattacharya and Petsche, 2005); on the other hand, non-artists showed more locally synchronized activities over prefrontal regions. Enhanced top-down control is also observed in the professional dancers during mental imagery of an improvised dance but not during a learned routine (i.e., classic waltz) (Fink et al., 2009a). On the other hand, during spontaneous musical improvisation, trained musicians show an activation pattern of a widespread neuronal network but with a simultaneous deactivation of dLPFC, a region usually involved in planning and conscious self-monitoring (Limb and Braun, 2008). Similar wide spread activation of brain network encompassing multitude of brain regions with a concomitant deactivation of dLPFC is also reported during the spontaneous lyrical improvisation in freestyle rap artists (Liu et al., 2012).

Altogether these studies suggest that creativity, either little-c or Big-C, cannot be localized to one or a few brain regions, rather they show that when humans are engaged with any sort of creative process, a multitude of brain regions become active,^2^ and these are also the brain regions that become active in many “ordinary” cognitive processes (e.g., working memory, attention, cognitive control, performance monitoring) which are not usually thought of as requiring creativity. Therefore, these studies help breaking the classical dogma that creativity is too complex or too difficult to study empirically, instead they suggest that creativity could be considered as a product of complex interplay between ordinary cognitive processes like memory, attention, executive function, and even emotion (Ward et al., 1999; Weisberg, 2006). However, the nature of interplay between the constituent cognitive (and affective) processes may not be trivial and the creative output can be considered as an emergent outcome of this intricate interplay, so a pure reductionist approach would not offer an adequate understanding of creative process(es).

### Remarks on neuroscience of creativity

As stated earlier, neuroscientific studies have contributed greatly to our understanding and subsequent demystification of creativity. However, one should also exercise caution while taking these neuroscientific findings into account, as there are several limitations and issues to consider. We do not provide here a detailed treatment on the scopes and limitations on possible interpretations of neuroimaging, but refer the interested readers to some recent monographs (Uttal, 2003; Satel and Lilienfeld, 2013); we highlight only some of the issues that are especially relevant for creativity research.

First, different neuroimaging techniques have different sets of assumptions, different spatial and temporal resolutions (e.g., excellent temporal resolution of EEG vs. slow blood-oxygen-level-dependent responses of f-MRI or poor localization of EEG due to ill-posed inverse problem vs. much higher spatial resolution of f-MRI), and therefore, tap different aspects of neuronal activities—e.g., EEG is a direct indicator of electrical activities, whereas f-MRI is an indirect indicator lacking a clear interpretation of neuronal origin (Logothetis, 2008). Hence, it is not surprising that a coherent picture of the neuroscience of creativity is yet to emerge; for a review see Dietrich and Kanso (2010).

Second, findings based on neuroimaging studies provide only correlational, but not causal, information, i.e., neuroscientific findings show that certain brain regions are correlated with creative tasks/measures under investigation but cannot prove whether these brain regions will be causally linked to creative task/measures. In this context, brain stimulation methods could provide some causal information, but evidence is still scarce (Cerruti and Schlaug, 2009; Chi and Snyder, 2011).

Third, brain responses related with a task are several magnitudes lower than those at rest, i.e., they are “noisy” and results are obtained after averaging across many trials and participants, so the neuroscientific findings are meaningful only in statistical sense. Therefore, it would be premature to claim that a brain region, location of which is obtained by statistical manipulations, is the seat of creative process(es). In fact, any search for finding an isolated seat of creativity in the brain is likely to be futile; it is the complex interplay between multiple and distant brain regions forming a network that is more likely to be associated with creative cognition (Bressler and Menon, 2010). One interesting proposal by Merker (2013) identifies not a locus of creativity (the dorsal pulvinar) but a potential locus of the selection process that is central to our model below. Further, we speculate that this network may be dynamically evolving as a creative process is not static, but a time-varying dynamic process (e.g., consider musical improvisation, “Because it’s improvised, musicians don’t know what they’ll play in advance; the notes emerge in the moment, from the complex give-and-take among the members of the ensemble‥‥ Creativity takes place over time, and most of the creativity occurs while doing the work” (Sawyer, 2012, p. 88), yet little is known about its time profile.

Fourth, the ecological validity of the most of the reported studies are necessarily compromised due to an overtly constrained environment in the laboratory (e.g., inside the scanner) and the impoverished nature of the adopted tasks, so the observed findings might not be generalizable to real-life creativity (Hasson and Honey, 2012). Further, most of these studies on creativity are based on samples of university students, though a common practice in the broad field of behavioral science, their generalizability may be quite limited (Henrich et al., 2010; Jones, 2010).

Finally, most of the neuroimaging studies on creativity have applied the subtraction method, i.e., by contrasting two conditions (i.e., creative vs. non-creative, insight vs. analysis, improvisation vs. memorization). A crucial assumption behind such a method is that the two conditions differ from each other in *only one* cognitive operation. This procedure is termed “pure insertion” because it assumes that the condition of interest is different from the other condition in the insertion of a single cognitive operation, and therefore, any differences in brain responses between the two conditions would be related to that single inserted operation. Though this assumption works well for perceptual or well-controlled cognitive task paradigms, it is difficult to achieve in task involving creative cognition as discussed below. For example, researchers often compared insight vs. analysis methods of problem solving based on a subjectively perceived “Aha!” response (Jung-Beeman et al., 2004; Kounios et al., 2006, 2008); yet the two methods differ on multiple cognitive processes including *impasse* (a state in which the solver is mentally stuck on an unsuitable construct of the problem and fails to progress further), *restructuring* (a mechanism by which the solver breaks out of mental impasse, and is a transition from an initial inappropriate and thus incorrect representation of a problem and state of not knowing how to proceed in solving a problem to a state of knowing how to solve it), and *deeper understanding* (a form of deeper or more appropriate understanding of the problem and its solution). Therefore, a straightforward comparison between insight and analysis method would contain more than one cognitive components, which implies that any neuroimaging finding on the basis of such comparison would be inadequate to isolate the unique neural component, if any, of insight. We believe this is a very serious limitation that deserves to be properly looked at before we can consider any claim of isolating neural correlates of creative process(es). Therefore, it is important to have a well-controlled task design so that the compared conditions in a neuroscientific study are as close as possible; see Weisberg (2013) for an elaborated discussion on this issue.

## A computational framework that accounts for creativity

We now propose an overarching, hypothetical, computational theory of creativity, which is a candidate formulation to help coordinate research in both psychology and neuroscience. It may serve as such because it is more detailed and mechanistic than most previous approaches, to the extent that it can be readily implemented on a computer. Implementations are made possible at multiple levels by application, in principle and practice, of the hierarchical approach of descriptive and explanatory modeling (Wiggins, 2007). It would be possible to proceed from this proposal either by attempting to falsify its various components (Popper, 1959), or testing the relationships between them, or by using it to identify particular areas of interest for future study (which also, of course, might ultimately lead to falsification). The theory is currently specified at a functional level considerably more abstract than that of neural activity, leaving scope for further theorizing at intermediate levels.

Our principal aim here is to propose an account of “spontaneous creativity”: the kind of creative cognitive events appear to happen spontaneously without any conscious volition. This kind of creativity is associated with the generation of language (Plotkin, 1998), music (Limb and Braun, 2008; Wiggins, 2012a), and along with others (Arieti, 1976; Loehle, 1994; Weisberg, 2006). We propose that it reflects spontaneous thinking and therefore represents a mechanism for domain-general creativity. The framework used is Baars’ Global Workspace Theory (GWT; Baars, 1988), enhanced with mechanisms based on Shannon’s Information Theory (Shannon and Weaver, 1949).

### Global workspace theory (GWT)

Bernard Baars (1988) has formulated the GWT, a theory of consciousness whose principal hypothesis is that conscious content must be globally available as it allows the content to be broadcast to many higher-level cognitive systems for possible motor control, executive functions, verbal awareness, and so on; in short, according to this theory, consciousness is equated to brain-wide information sharing (Dehaene, 2014). The theory posits a framework within which consciousness can take place, based around a multi-agent cognitive architecture (Minsky, 1985) communicating via something like an AI blackboard system (Corkill, 1991), but with constraints, outlined below. We avoid Chalmers’ “hard” question of “what is conscious?” (Chalmers, 1996) and instead ask “what is it conscious of, and how?” This is appropriate, because the nature of consciousness is not our central issue, but presentation of information to it is.

Baars models the non-conscious mind as a large collection of expert generators, like the multiple experts in Minsky’s (1985) *The Society of Mind*, but with less clear hierarchy, processing information in massive parallel, competing for access to a Global Workspace via which (and only via which) information is exchanged; information must cross a notional threshold of importance before it is allowed in, and we return to this below. The Global Workspace is visible to all generators, and contains the information of which the organism is conscious at any given time. It is capable of containing exactly one “thing” at once, though the nature of that “thing” is underspecified. It is highly contextualized, and meaning in it is context-sensitive and structured; contexts can contain goals, desires, etc. Baars mentions the possibility of creativity within this framework in passing, implicitly equating entry of a generator’s output into consciousness with the “Aha!” moment (Wallas, 1926), but does not develop this idea further.

Baars proposes that information integration happens sequentially, i.e., in stages, via something that one might (but he does not) call local workspaces, that integrate information step by step in a sequence, rather than all in one go as it arrives in the Global Workspace. This information integration approach has been extended later by Tononi and Edelman (1998), who propose information-theoretic measures of information integration as a measure of consciousness of an information-processing mechanism. Baars has embraced the information-theoretic stance, too, and the three authors have jointly proposed to begin implementing a conscious machine (Edelman et al., 2011) based on their ideas. The current work may contribute to this endeavor, though probably at a level more abstract from neurophysiology than these authors intend.

### The threshold paradox

**Figure 1:**
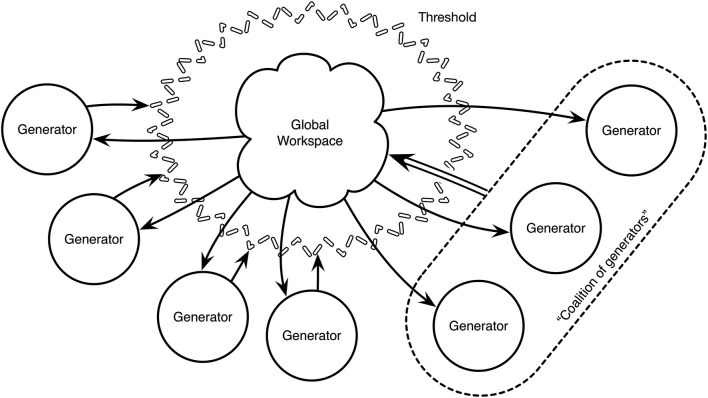
**Illustration of Baars’ threshold paradox**. Generators operate on perceptual input and associative memory. In order to reach into the level of consciousness, a coalition of generators needs to be formed and this is possible only via the Global Workspace. However, before it can be made possible, support from the generators that are to be recruited are needed, and therein lies the paradox.

Baars (1988, pp. 98–99) also addresses what he acknowledges is a problem for his theory. He proposes a threshold for input access to the Global Workspace, crossing of which is thought of in terms of “recruiting” sufficient generators to produce information that is somehow coordinated, or synchronized between them. However, in terms of the Global Workspace alone, there is no means of doing this: generators can only be coordinated (whatever that means) via the Global Workspace, and so the generators are faced with a classic Catch 22 situation. This form of the Workspace is illustrated in Figure 1. Baars presents two possible solutions to this paradox, but both are somewhat incomplete, therefore causing a gap in the theory. Our approach presents a possible solution, and simultaneously accounts for the “Aha!” moment.

### Perception, anticipation, and evolution

#### Reaction vs. anticipation

We now present a mechanism for managing the competition between generators in Baars’ system, after Wiggins (2012b). The key distinctions are: (a) between the information content and entropy (defined below) of various stimuli; and (b) between organisms that react and organisms that anticipate. Our model is inspired by evolution after taking into consideration of the evolutionary advantage conferred by the resulting behaviors. Thus, the evolutionary argument presented here is part of the design, and not merely an example.

In artificial intelligence, an agent (of which an creative agent is presumably an instance) is defined as a program or robot with a perception-action cycle: its action is purely shaped by its perceptions of the environment or world. Lower organisms seem to be modeled well by such simple agents, as their actions are limited to reacting to environmental conditions, coping rather poorly when their evolved reactive program is interrupted or the environment is suddenly altered. However, to model higher cognitive development, one could consider a more predictive system as opposed to a reactive one; here an organism is in predicting mode perpetually as it forms the prediction from a learned model of previous sensory data, what is likely to come next, and compared this with current sensory input. A related mechanism based on expectation and prediction was proposed by Sokolov (1963) on orientation reflex, and there is currently increasing interest in predictive coding in perception; Hohwy (2014) gives an excellent philosophical introduction to predictive coding. The principal advantage of such anticipation is a simple mechanism for spotting what is unusual, what, therefore, constitutes a potential new opportunity or threat, and what deserves cognitive resource, or attention. Our proposal draws together orientation reflex and predictive coding of perceptual input into one process of information management and attention resource deployment, and suggests how creativity can be a by-product of these two related things.

#### The consequence of sequence: managing uncertainty with expectation

The most important feature of an autonomous agent is that it can predict what is to come next, and react, or prepare to react, in advance. To predict usefully in a changing world, an organism must learn. It must be able to learn not just categorizations (to understand what something is), but also associations (to associate co-occurrence of events with reward or threat), and, crucially here, *sequence*.

However, a simple statistical learning mechanism is not subtle enough (Huron, 2006). Evolutionary success entails that an organism breeds, so learning only from potentially fatal consequences will not do: if the experience kills the organism, there is little benefit of that experience. The successful strategy here is at a meta-level, above the learned body of experience: if an organism is aware that it is in circumstances where its predictions are uncertain, it must behave more cautiously, its metabolism should be prepared for flight, and it should devote extra attention to its surroundings. Huron (2006) suggests that this effect serves as an exaptation or spandrel producing part of the affect of music; but, here, the mere adaptation suffices: self-evidently, uncertainty affects behavior in humans and other animals, and doing so does not rely on explicit reasoning. Indeed, the converse is true: we feel nervous in uncertain situations, and the feeling makes us pay attention to appropriate sensory inputs and prepare for flight. This mechanism, and the associated affective response, is not the same as fear, but can lead there in extremis.

Finally, any kind of learning of this nature must include *generalization*—from both co-occurrence and sequence—that similar consequences arise from similar events, encounters, etc. Without this, mere tension cannot lead to fear at the sight of the bared fangs of an unknown but large animal. This accords with Gärdenfors’ (2000) proposal, that perceptual learning systems are motivated to understand similarities and differences between perceived entities in the world, and to place observations at the appropriate point with respect to previous experience.

#### Prediction and selection

Given a world-model, categorized into types, situations, etc., a set of generators with recent and current perceptual inputs matched against precursors of sequential associations, can make stochastic predictions conditioned by prior observations. Making predictions quickly, sequentially, would be valuable, but slow, multiple predictions, in parallel, are a more likely candidate for evolutionary success, and the more the better—as in Baars’ proposal. But many predictions occurring simultaneously will be chaotic and disorganized, so how will useful candidates for prediction be selected? Baars’ solution is the problematic threshold, described above.

The GWT is unclear about the precise notion of generators “recruiting” each other. The desired effect is something like an additive weight: the more generators “recruited”, the greater their impact. We will avoid answering this question, by approximating this effect probabilistically.

Our proposal is based on statistical, frequentist notions of learning, and so our account is in terms of statistical models; however, we do not claim that the reasoning is in principle exclusive to such models. In this view of the system surrounding the Global Workspace, there are many independent subsystems, making multiple predictions from a predictive (statistical) model of (assumedly) reasonable quality. We must assume imperfect models: each generator can only have a partial view of its world and its predictions, since modeling everything all the time would be prohibitively expensive. It follows from the use of statistical models that the more expected occurrences are the more likely ones to be predicted. Conversely, extremely unlikely predictions will rarely be “popular”.

In a model of prediction and action based only on observed frequency, an organism will do the commonest thing, even if inappropriate, and so will fail: it will not predict unlikely or surprising situations, and will not prepare itself against eventualities.

In Baars’ theory, the most likely outcome, corresponds with multiple generators “in coalition” generating that outcome. The likelihood of each generator predicting an outcome is proportional to the popularity of that outcome across the set of generators. Thus, we can use the likelihood of the outcome, *p*, to model its popularity.

In reality, though, we know well that an animal will not just carry on as normal in unexpected circumstances: it will experience the negative-affective response, described above, so we need a mechanism for that response. The obvious choice is the notion of *entropy*, formalized by Shannon (1948). MacKay (2003) makes a finer distinction between *information content*,*h*, defined as an estimate of the number of bits required to describe an event, *e*, in a context, *c*, denoted by (*c*|*e*), or its *unexpectedness*:

(1) $$h\left(e|c\right)=-\log_{2}p\left(e|c\right)$$

and entropy, *H*, defined as an estimate of the uncertainty inherent in the distribution of the set of events *E* from which that *e* might be selected, given the context, *c*:

(2) $$H\left(c\right)=\sum_{e\in E}p\left(e|c\right)\;h\left(e|c\right)=-\sum_{e\in E}p\left(e|c\right)\log_{2}p(e|c).$$

*H* is maximized when all outcomes are equally likely, and minimized when a single outcome is certain. Both *h* and *H* are useful to our hypothetical animal, but here we consider only *h*.

*h_t_* is the unexpectedness of a partial model of the actual on-going experience in a particular state, *t*. If the experience is likely (that is, if it is predicted as likely from what has gone before), it is not unexpected, and therefore *h_t_* is low; on the other hand, if the experience is unlikely, it is unexpected, and so *h_t_* is high. In pure frequentist terms, a completely new experience is maximally unlikely. To model this, we propose that the selection process is sensitive to *h_t_*, and decreases priority of generators when it is low. Thus, the likelihood of models of the experience in which the new scent is included being accepted in the Global Workspace is positively related to its unexpectedness. We call this the *recognition-h* case; it explains why unexpected events attract attention.

Now, consider *h*_*t*+1_, the unexpectedness of a predicted situation: the *prediction-h* case. It is maximally unlikely that a prediction will be made including a previously unencountered scent, so we would expect *h*_*t*+1_ to be high, causing alarm. Excess of such predictions, or repeated occurrence of a single one, would lead to a state of constant anxiety.^3^ This explains why surprising (or interesting) predictions are more likely to draw attention than prosaic ones.

Of course, in a simplistic frequentist account, predictions introducing new percepts or concepts cannot arise, because they entail the creation of new symbols. This is why it is necessary to include generalization and/or interpolation in the theory (see above).

**Figure 2:**
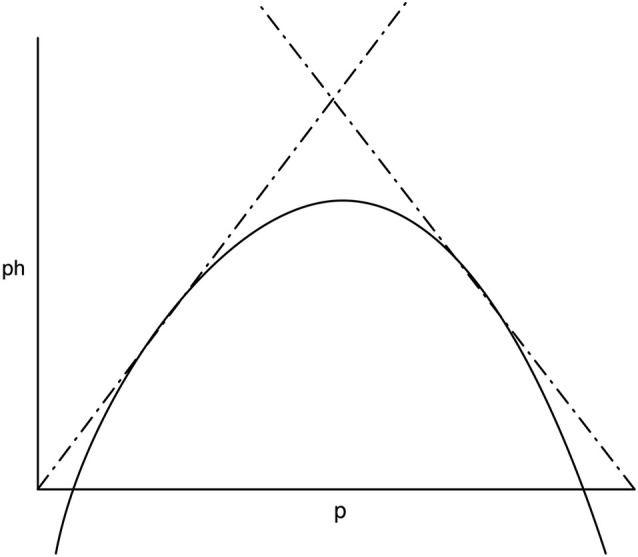
**Illustration of the interaction between likelihood and unexpectedness.** The overall likelihood (solid) is formed by the multiplication of two monotonic functions: the unexpectedness of a generated item (dashed) and the number of generators likely to agree on it, according to its likelihood (dotted).

The problem of over-active prediction-*h* is mitigated by the mechanism supplied above, in which prediction is probabilistic and (broadly) additive across predictors, modeled by *p*. There are two opposing forces here, one of which changes inversely relative to the other, and because they are co-occurrent, their effects should (broadly) multiply. Therefore, the overall popular outcome among the generators in the global workspace can be estimated by multiplying the probability, *p* of an event (which estimates the likely number of generators predicting it) by *h* (which estimates the volume at which they are predicting). The resulting likelihood is illustrated by the unit-free diagram in Figure 2. It biases away from predictions which are either very likely or least expected, reducing the power of very unlikely or very obvious predictions to attract attention. This may explain why unlikely possibilities do not prevent action by overwhelming the acting organism with choice.

Recognition-*h* and prediction-*h* are quite different in the context of the Global Workspace. We propose that generators may generate structures of either kind, and that the two *hs* will compete for the resource of attention. In this way, present danger or benefit outweighs predicted likelihoods, because the distribution of potential predictions is over a much wider range of possibilities than that over actual perceptions, and so probability mass is spread more thinly. Conversely, for example, likely but unexpected predicted benefits can outweigh less seriously dangerous present circumstances—thus, prioritizing an unusual opportunity can be mechanistically explained as an emergent behavior. As there are two kinds of generation, we must propose a means of distinguishing between them: otherwise, consciousness could not distinguish between the perceived and the predicted world.^4^

**Figure 3:**
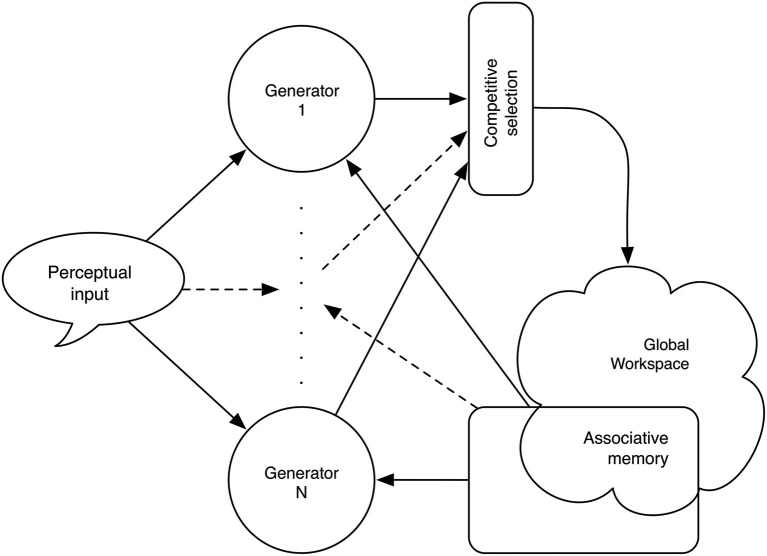
**Schematic diagram of Wiggins (2012b) proposal for the Global Workspace**. In this version, there is no need for a threshold of access. Instead, the generators compete one against another and probability and information content determine the winner.

At any given moment, this “popularity” value, *p × h*, is used in deciding which of the range of possible inputs, derived from matching sensory input to statistical models in memory, enters the Global Workspace. This is illustrated in Figure 3.

### Creativity from prediction

The remaining question is now: how does this mechanism for choosing access to consciousness help to understand and simulate creative cognition? We propose that a surprising answer may be found in the opening sentence of Mozart’s comment, above, which might be paraphrased as “when I am not being bothered, and when I have no worries and no particular goals”, which in turn means “when I have no distractions” or “when I have no information-rich input to consciousness from outside or within”. In this situation, the Global Workspace is occupied only by weakly-informative ephemera, and its generators are receiving little or no external stimulus.

Earlier we proposed that anticipatory animals base their actions not on stimuli, but on the results of comparing stimuli with predictions about the state of the world made from previous state(s). We now propose that those generators continue to generate at all times. Generators can freewheel, within the same statistical framework as above, but lacking the statistical influence of an external stimulus. The outputs will be more diffuse than when directly stimulated, but they can still enter the Global Workspace, in the absence of competition. Given this, the diagram in Figure 2 can be seen as the Wundt curve (Wundt and Titchener, 1904; Margulis and Beatty, 2008), as it defines a sweet spot of balance between dullness and over-complexity in information-theoretic terms.

The mechanism above also accounts for why a stimulus of one type may give rise to a creative production of another: the perception conditions the sampling, and this affects the likely outcomes, which are generated all the time. The ones with the right statistical properties make it into the Global Workspace, and can thence be further elaborated.

The mechanism works with any model from which meaningful statistical likelihoods can be estimated. Therefore, it can account for the generation of sentences, and possibly internal speech, commonly equated with essential thought. Therefore the current approach can account for general creative thought and for the emergence of particular thoughts into consciousness as intuition.

At this point, we have dispensed with an explanation of creativity as a special mechanism: in our approach, non-conscious creativity is happening continually as a result of on-going anticipation in all sensory (and other) modalities. When conditions are right, this essential survival mechanism gives rise to creativity as a side effect.

## Relating neurophysiology to the cognitive architecture

The cognitive architecture presented in the previous section is located at a very abstract level, distant from neural models. Nevertheless, some success has been achieved by neurophysiological and behavioral methods, relating the information-theoretic signals (i.e., the statistical values that change in time) with measurable responses (Pearce, 2005; Pearce et al., 2010a; Hansen and Pearce, 2012; Egermann et al., 2013). With such empirical grounding, the possibility of filling in the gaps in a methodical way becomes available.

Here, we relate the proposal above with other cognitive and neurophysiological theories of creativity. Recall the distinction introduced earlier between spontaneous creativity and creative reasoning. These, respectively, correspond with non-conscious and conscious creative activity, the point of transition from the former to the latter corresponding with Wallas (1926) “Aha!” moment, illumination. We briefly focus on three related aspects here: unconscious thought, mind wandering, and spontaneous intrinsic brain activities. We argue that non-conscious creativity happens prior to conscious creativity and this distinction is exemplified in detail by Wiggins (2012a). In context of GWT, the “Aha!” moment is the entry of an idea into the Global Workspace; ideas must compete in relative terms, and the winners are the ones that enter consciousness (Wiggins, 2012a).

### Unconscious/conscious processing in creativity

It has been widely accepted that creativity cannot solely be explained by conscious processes alone; conscious thought has limited processing capacity (Miller, 1956), yet unconscious thought (i.e., being distracted yet still actively maintaining a task-related goal in the background) could process a vast amount of information (Dijksterhuis and Nordgren, 2006; Ham et al., 2009; Lerouge, 2009). Earlier spontaneous unconscious thought is shown to benefit complex decisions requiring manipulation of multiple attributes (Dijksterhuis et al., 2006). Here, participants were given various alternatives with the aim to choose the most attractive option; participants chose either immediately or after a period of deliberate thinking or after performing a distracting task. It was found that participants who performed a distracting task actually made a better and more optimal decision than those who performed conscious deliberation before taking the final decision. Interestingly, this beneficial effect of unconscious thought holds only for complex decisions, while conscious deliberation outperforms unconscious thought for simple decisions (i.e., decisions involving only a few attributes) (Dijksterhuis and Nordgren, 2006; Dijksterhuis et al., 2006). Since the selection of a creative idea among many possible alternatives can be considered a decision making process, unconscious thought may have a beneficial effect on the idea selection phase of the creative process. If this to be the case, it party explains why there are many anecdotal evidence of unconscious processing in real life creativity focusing on idea selection (Ghiselin, 1952), whereas the empirical studies focusing on idea generation provide a weaker evidence (Ritter et al., 2012).

In creativity, the time period during which the unconscious mental processes are “active” is termed “incubation”(Wallas, 1926), and one direct way incubation helps problem solving is by reducing mental fatigue (Kahneman, 1973). Further, the mental set-shifting hypothesis suggests that putting an unsolved problem aside for a while and then coming back to it would help eliminating incorrect old representations leading earlier to mental fixedness, and allowing the emergence of the most optimal or correct representation (Schooler and Melcher, 1995). However, one could argue that this primarily demonstrates a passive role for unconscious thought, on the other hand, unconscious deliberation can also be assumed to be a proactive and goal-driven process (Dijksterhuis and Nordgren, 2006; Bos et al., 2008), and recent findings do provide evidence in favor of the latter account. For example, in the study of Dijksterhuis and Meurs (2006) participants had to list Dutch places names starting with the letter “A” or letter “H”, and it was found that the participants who were engaged with conscious deliberation listed more names of large cities and towns, where as the participants who were engaged with unconscious thought listed more names of small villages. This demonstrates that unconscious thought facilitates access to unconventional or non-dominant information in the long term memory, thereby, potentially promoting remote creative association. This possibility is indeed supported by Zhong et al. (2008) who found that participants engaged with unconscious thought were faster to solve difficult RAT problems than participants who were engaged with conscious thought. Interestingly, the number of solved problems that were very difficult did not differ significantly between the two thought conditions, but conscious thought led to more solutions of easy problems than unconscious thought. This facilitatory effect of unconscious thought in solving complex tasks including creative problem solving is explained by a two-stage processes (Zhong et al., 2008) that is very similar to our earlier proposition (Wiggins, 2012b): the first stage involves unconscious deliberation generating creative ideas by “deep cognitive activation” (Wegner and Smart, 1997) and the second phase is the transfer of output from the unconscious to conscious awareness; an incomplete processing at either step would impair the influence of unconscious thought on creativity.

### Mind wandering in creativity

A closely related mental phenomenon is mind wandering, also known as task-unrelated thought. Mind wandering, a sort of day-dreaming, involves a shift of attention away from a primary task to process some other, personal information, but in a way that is not obviously goal-directed. The major characteristic of mind wandering is that the attention is decoupled from the immediate task context (Schooler et al., 2011), which allows the mind to shift towards cognitions unrelated to the current demands of the external environment (Singer, 1966). Mind wandering is a very common process: experience sampling studies suggest that up to 50 percent of our waking thought is stimulus/task unrelated (Killingsworth and Gilbert, 2010). Mind wandering occurs without intention or even awareness (Smallwood and Schooler, 2006), and when mind wanders, people are often not aware of their thoughts.

Although there are several costs of mind wandering including a failure of cognitive control (see Table 1 of Mooneyham and Schooler, 2013), several new lines of research suggest that mind wandering could be linked to creative thinking. For example, individuals with attention-deficit/hyperactivity disorder (ADHD), which is known to be associated with high level of mind wandering, tend to score higher than individuals without ADHD on laboratory measures of creativity (White and Shah, 2006). Zhiyan and Singer (1997) reported that positive constructive daydreaming, a style of daydreaming associated with playful and constructive imagination, is correlated with openness to experience, a personality trait correlates positively with creativity (McCrae, 1987). Mind wandering is also assumed to be linked to the periods of incubation and insight of the creative process. A recent meta-analysis (Sio and Ormerod, 2009) suggests that incubation is most effective when the incubation period is filled with an undemanding task as compared to demanding task or a no task at all. Although mind-wandering seems to be spontaneous and a resource-free process, it is, in fact, supposed to be a resource intensive process (Smallwood and Schooler, 2006) (but see also McVay and Kane, 2010); therefore, any demanding task that tax working memory decreases mind wandering, and conversely, an undemanding task on working memory promotes mind wandering. Recently, Baird et al. (2012) combined these two lines of evidence by showing that the benefits of incubation intervals are greater in divergent thinking tasks when participants were distracted by an undemanding task than when they were engaged with either a demanding task or no task at all. As the mind wandering is more frequent in undemanding tasks than the two other conditions, this result shows that one feature that may characterize successful incubation intervals could be the opportunity of the mind to wander. It should be noted, however, that the reported beneficial effect is only found for the previously presented tasks but not for the new ones, suggesting that the relationship between mind wandering and creativity cannot be easily generalized, but could be mediated by several factors, like contents and durations of mind wandering, working memory capacity, personality traits, and dimensions of creativity (e.g., novelty, appropriateness).

In a seminal study using f-MRI, Mason et al. (2007) showed that periods of mind wandering correlate with activity in a constellation of neural regions across the brain, known collectively as the default network (Raichle et al., 2001). The default network is a network of cortical and subcortical structures (including the anterior and posterior cingulate cortices, precuneus region, the medial prefrontal cortex and the posterior parietal lobule) (Raichle et al., 2001; Buckner, 2012); this network is particularly activated when participants are at rest and deactivated when engaged with demanding tasks with high central executive demand. This further supports the notion that mind wandering is inversely related with cognitive demand and associated with a reduced cognitive control and a broadening of attention (Antrobus et al., 1966; Antrobus, 1968). Interestingly, a subsequent study by Christoff et al. (2009) found that in addition to the default network, mind wandering is also associated with executive network (lateral PFC, inferior parietal lobe), and the activations of these dual, apparently functionally opposing, networks occur for those mind wandering without metacognition. This finding is intriguing because the executive network is usually antagonistic to the default network (Fox et al., 2005): when one network is activated, the other is deactivated; hence, mind wandering without awareness seems to be a unique mental state that allows co-activations of these two opposing brain networks.

Therefore, mind wandering can also be considered as a goal-driven process despite the fact that it is not explicitly directed towards an internal task (Smallwood and Schooler, 2006), and has an access to the same global workspace during internally generated thought (see Smallwood et al., 2012 for a review).

### Spontaneous neuronal activity: cradle of creativity?

The suggestion of ideas competing for entry to conscious awareness entails the suggestion of multiple ideas being produced, and some falling by the wayside. Such a situation is entirely alien to conscious personal experience, but that is what we would expect: one should not be conscious of information that fails to enter one’s consciousness. But what evidence is there of such activity?

As discussed earlier creative thinking may be spontaneous, sudden and without any conscious forewarning; it can occur with or without external input, and is often the product of long labor of unconscious efforts preceded by a mental impasse out of focused efforts. Insight is not necessarily complete at first, so must be subsequently improved. Brilliant ideas must be worked for, and worked after! Little is known about the underlying spontaneous brain mechanisms of such creative processes; however, short periods of disengagement or rest may increase the likelihood of a flash of insight, i.e., the “resting brain” could be conducive for creative thinking. Earlier we have discussed the roles of unconscious thought and mind wandering in creative cognition. Here we suggest that the mechanism by which the “best idea” enters into the realm of consciousness may be visible in spontaneous neuronal activity, i.e., resting state brain activity (Raichle and Mintun, 2006). Although the neuroscientific research on creativity is mostly concerned with brain responses during the performance of a creative task, we suggest that it is in the spontaneous and dynamic fluctuations of brain activity patterns that the seed of creative cognition may reside.

However, the brain is never actually at rest: its energy consumption in a spontaneous/resting state is much larger compared to the additional energy consumption during cognitive tasks (Raichle, 2010). Earlier we highlighted the default mode network (Raichle et al., 2001) that becomes more activated during the resting or inactive state than during the performance of a task. Interestingly, the concept of such network was first put forward by a noted creativity researcher Nancy Andreasen et al. (1995) who noted that “free-ranging mental activity (random episodic memory) produces large activations in association cortex and may reflect both active retrieval of past experiences and planning of future experiences”,; the “rest” is then appropriately rephrased by “Random Episodic Silent Thinking” and reflects ongoing spontaneously occurring long-term memory retrieval and encoding (Andreasen et al., 1995). Binder et al. (1999) showed that for perceptual tasks the default network gets attenuated, and for conceptual tasks, the default network is activated much as in the resting state, and noted that default networks are “active during conscious resting and are engaged in such processes as retrieval or information from long-term memory, information representation in conscious awareness in the form of mental images and thoughts, and manipulation of this information for problem-solving and planning” (Binder et al., 1999, p. 86); this crucially links with our earlier discussion on mind wandering and internally generated thought processes. Further, this ability of building alternative mental models and of simulating future scenarios as possibly mediated by the default network, often processed at the subconscious level (Buckner and Caroll, 2007), are considered to facilitate real-life problem solving and creativity (Hogarth, 1987; Antonietti, 1991).

The default network is also of interest to creative processes for various reasons. The default network regions (anterior cingulate and posterior cingulate cortices) are found to be more active immediately before the presentation of RAT problems solved with subjectively reported insight as compared to those solved without insight (Kounios et al., 2006). Further, some default network regions are also activated during a creative story generation from a list of unrelated words (Howard-Jones et al., 2005). Recently, Ellamil et al. (2012) asked participants to design book cover illustrations while allowing switching between the generative and exploratory stages of the creative process, and they found more activations in default network regions (medial PFC, posterior cingulate, temporoparietal junction) during creative evaluation process as compared to creative generation process. Takeuchi et al. (2011) found an enhanced activation of the precuneus, a core component of default network, with heightened creative performance in an effortful working memory task. Recently the resting state functional connectivity analysis showed that the strength of correlation between the medial PFC and the posterior cingulate, two core default regions, is positively associated with scores on DTT (Takeuchi et al., 2012). Further, the resting-state network fluctuations differentiate personal styles of problem solving, i.e., insight vs. analytic (Kounios et al., 2008).

### Prediction and empirical validation

The level of detail inherent in our proposed research program will allow formulation of specific and testable hypotheses, and more exploratory work, such as our search for neural correlates of information content signals (Pearce et al., 2010a). What is more, the possibility of computational implementation admits more rigorous testing of the theory than is available with pencil-and-paper models. Thus, a program such as that proposed above may assist neuroscientific study by providing a hypothetical map of the territory. Then, work may be focused in such a way as to test structural hypotheses efficiently and quickly, either falsifying the framework or allowing it to develop into a Lakatosian core (Lakatos, 1978). What is more, computational and behavioral methods can be applied to the same program, not only testing its formulation further, but also uniting computational, behavioral and neuroscientific thinking at multiple levels.

Our framework predicts certain specific perceptual events in conscious experience—most obviously, the time-variant information-theoretic signals that filter items into consciousness. We have shown empirically that, in some circumstances, these perceptual events correlate with particular measurable electrophysiological events in the brain (Pearce et al., 2010a). Therefore, the current proposal raises the possibility of multi-faceted empirical attack on the problem of ideation by means of simulation, prediction, and empirical validation/falsification. For example, in our work on segmentation (Pearce et al., 2010b), we have demonstrated that bottom-up information-theoretic predictions are at least as reliable as top-down rules in simulating human chunking of musical sequences. The framework proposed here can in principle account for this, and related linguistic effects (chunking and “garden-path” sentences) in terms of competition between alternative hypothetical chunks, and we are currently empirically testing of this account.

## Conclusion

In this paper, we have argued for a tripartite research program for the neuroscience of creativity, based around: (i) computational modeling; and (ii) behavioral confirmation. We have suggested that the many various attempts at mapping, of high quality though they be, risk degeneration into a directionless activity without overarching theories of the cognitive function that is associated with them. We have proposed a computational modeling paradigm of creativity by extending Baars’ GWT based on the principles of information theory, and we have touched on current work to examine the predictions of our approach. The whole, we suggest, can only be empirically stronger than the sum of its parts, and such strength is required to address the difficult and fundamental question of creativity—which, after all, is part of what it means to be human.

## Conflict of interest statement

The authors declare that the research was conducted in the absence of any commercial or financial relationships that could be construed as a potential conflict of interest.

## Footnotes

^1^At least, if we eschew descent into mysticism.

^2^By “active”, we mean some sort of involvement or association of brain regions in specific creative process(es), since brain areas are shown to be both positively (activated in neuroimaging sense) and negatively (deactivated) correlated with creativity.

^3^Some root symptoms of clinical anxiety would be explicable in terms of a breakdown of this mechanism.

^4^Coupled with a deficit in suppression of less likely outcomes, as above, this situation might lead to some root symptoms of schizophrenia: hallucinations, delusions, and cognitive distortions.
